# 48019 Create a mouse model of chronic sleep deprivation by specific-neuron targeted ablation

**DOI:** 10.1017/cts.2021.439

**Published:** 2021-03-30

**Authors:** Toshihiro Imamura, Allan I. Pack

**Affiliations:** 1 Children’s Hospital of Philadelphia; 2 University of Pennsylvania

## Abstract

ABSTRACT IMPACT: A mouse model of minimally-invasive chronic sleep deprivation is essential for elucidating the impact of sleep deprivation on various health issues, and it would lead to the possibility of sleep as a therapeutic target. OBJECTIVES/GOALS: The lack of sleep has been associated with various health conditions. In mice, sleep deprivation has been achieved mainly by physical disturbances, which raises concern about confounding effects by stresses. Without physical disturbance, targeted neuron ablation can address this methodological flaw. METHODS/STUDY POPULATION: AdultVgat-IRES-cre mice undergo a stereotaxic injection of adeno-associated virus (AAV) vector containing mCherry-dtA to bilateral parafacial zone (PZ) to perform GABAergic neuron-specific cell ablation. Control mice receive an injection of AAV vector containing hSyn-DIO-mCherry. All mice are implanted with electroencephalogram and electromyogram (EEG/EMG) electrodes for sleep-wake analysis. After 7-10 days of the postoperative recovery period, mice are kept individually in a cage for sleep-wake state recording. EEG/EMG and video recording are used to measure total wake time, total sleep time, percent of rapid eye movement (REM) and non-REM sleep, and detailed characterization with spectral analysis. RESULTS/ANTICIPATED RESULTS: We anticipate that the ablation of GABAergic neurons in bilateral PZ decreases the fraction of sleep state in mice, especially non-REM sleep. In the Vgat-IRES-cre mice that received the injection of AAV vector containing mCherry-dtA, total sleep time is expected to be decreased constantly during the 8-week observation period. Sleep-wake staging by video activity recording is anticipated to be closely correlated with the gold standard staging by EEG/EMG. Possible stresses caused by the restriction of physical activity and handling of mice for EEG/EMG recording can be further minimized by the sleep-wake staging performed with the video activity recording. DISCUSSION/SIGNIFICANCE OF FINDINGS: The lack of sleep has been associated with negatively affecting overall health and is implicated in major health conditions including obesity, diabetes, and cardiovascular diseases. This chronic sleep deprivation mouse model can be used to understand the mechanisms of such detrimental effects on health, and would improve many health conditions.

